# Motion artifact reduction for magnetic resonance imaging with deep learning and k-space analysis

**DOI:** 10.1371/journal.pone.0278668

**Published:** 2023-01-05

**Authors:** Long Cui, Yang Song, Yida Wang, Rui Wang, Dongmei Wu, Haibin Xie, Jianqi Li, Guang Yang

**Affiliations:** Department of Physics, Shanghai Key Laboratory of Magnetic Resonance, East China Normal University, Shanghai, China; Sun Yat-Sen University, CHINA

## Abstract

Motion artifacts deteriorate the quality of magnetic resonance (MR) images. This study proposes a new method to detect phase-encoding (PE) lines corrupted by motion and remove motion artifacts in MR images. 67 cases containing 8710 slices of axial T2-weighted images from the IXI public dataset were split into three datasets, i.e., training (50 cases/6500 slices), validation (5/650), and test (12/1560) sets. First, motion-corrupted k-spaces and images were simulated using a pseudo-random sampling order and random motion tracks. A convolutional neural network (CNN) model was trained to filter the motion-corrupted images. Then, the k-space of the filtered image was compared with the motion-corrupted k-space line-by-line, to detect the PE lines affected by motion. Finally, the unaffected PE lines were used to reconstruct the final image using compressed sensing (CS). For the simulated images with 35%, 40%, 45%, and 50% unaffected PE lines, the mean peak signal-to-noise ratio (PSNRs) of resulting images (mean±standard deviation) were 36.129±3.678, 38.646±3.526, 40.426±3.223, and 41.510±3.167, respectively, and the mean structural similarity (SSIMs) were 0.950±0.046, 0.964±0.035, 0.975±0.025, and 0.979±0.023, respectively. For images with more than 35% PE lines unaffected by motion, images reconstructed with proposed algorithm exhibited better quality than those images reconstructed with CS using 35% under-sampled data (PSNR 37.678±3.261, SSIM 0.964±0.028). It was proved that deep learning and k-space analysis can detect the k-space PE lines affected by motion and CS can be used to reconstruct images from unaffected data, effectively alleviating the motion artifacts.

## Introduction

Magnetic resonance imaging (MRI) is one of the most widely used medical imaging techniques. It not only features high resolution and good contrast for soft tissues, but also is noninvasive and safe. However, the MRI technique is also complex, and is vulnerable to various types of image artifacts. Among these artifacts, motion artifacts caused by voluntary or involuntary motions of a patient may be the most common, owing to the long scan time. As motion artifacts can seriously impair image quality and can even make images non-diagnostic, the alleviation of motion artifacts has always been a popular topic in MRI.

Both prospective and retrospective approaches have been proposed for suppressing motion artifacts [1], and most of these approaches can alleviate the motion artifacts to a certain extent in certain cases. However, there seems to be no "silver bullet" for motion artifact correction in MRI [1].

The most straightforward and effective way to avoid motion artifacts is to reconstruct images with data that was not affected by motion [1]. By using fast MRI sequences, the scan time can be significantly reduced, reducing the chances of patient motion, and thereby suppressing motion artifacts. Compressed sensing (CS) [2] can also be used to shorten the scan time. It collects a part of k-space data with a pseudo-random sampling scheme, and reconstructs a high-quality MRI image from the under-sampled data using sparse constraints and nonlinear algorithms. Among the different CS algorithms, the split Bregman algorithm [3] is superior to the other algorithms in terms of reconstruction speed. Compared with full-sampled images, images reconstructed with CS normally still lose some image details, and are slightly blurrier [4], however, CS has already been widely adapted in commercial MRI scanners because of meeting the requirement of clinical use from under-sampled k-space data.

A convolutional neural network (CNN) [5] has been successfully applied to the inverse problems of image denoising and reconstruction [6]. It has also been used in MRI reconstruction, achieving good results in terms of image quality and reconstruction speed [7–9]. Compared with the iterative reconstructions of CS, the CNN model training takes a very long time, however, the image reconstruction time when using a trained CNN is shortened considerably, to the extent of real-time reconstruction [10]. Thomas Kustner [11] used a CNN to detect and quantify motion artifacts in MR images. In some studies [11–13], CNN were used as quality assurance approach to detect and abandon the images with motion artifacts, existing the disadvantage of rescanning to acquire images with high quality, which longer scanning time has to be required. Haskell, MW, et al. [14] combined a CNN model with a model-based data-consistency optimization to mitigate motion artifacts, and found that using the CNN alone can mitigate motion, but at the expense of introduced blurring. CNN models have also been used as generators of types of generative adversarial network (GAN) to remove motion artifacts well [15–17], however, it was easy to suffer from over smoothing, which lost diagnostic information.

In this paper, we propose a new approach for removing motion artifacts and obtaining high-quality MR images. First, we trained a CNN model to filter a motion-corrupted image, and to partially remove motion artifacts. Then, we compared the k-space of the filtered image with the motion-corrupted k-space to determine the k-space lines not affected by the motion. Finally, those lines were used as the under-sampled data from which the final image was reconstructed, using the split Bregman method.

## Material and methods

### Data information

We used 67 cases, consisting of 8710 total slices of axial T2-weighted images from the IXI (http://brain-development.org/ixi-dataset/) public dataset (IXI-T2). They were all collected by Hammersmith Hospital from the normal and healthy subjects using a Philips 3.0 T system with the scanner parameters as follows: repetition time = 5725.790ms, echo time = 100.0ms, number of phase encoding steps = 187, echo train length = 16, reconstruction diameter = 240.0mm, Acquisition Matrix = 192 × 187, and flip angle = 90 degrees. These were all magnitude images, without distinct motion artifacts. The image resolution was 256 × 256 × 130(height × width × slice) and the voxel size was 0.9375mm × 0.9375mm × 1.25mm. For training the CNN model to remove motion artifacts, 6500 slices from 50 cases were used as a training set, 650 images from 5 cases were used as a validation set, and 1560 images from 12 cases were used as a test set.

### Synthesis of motion-corrupted images

**Fig 1:**
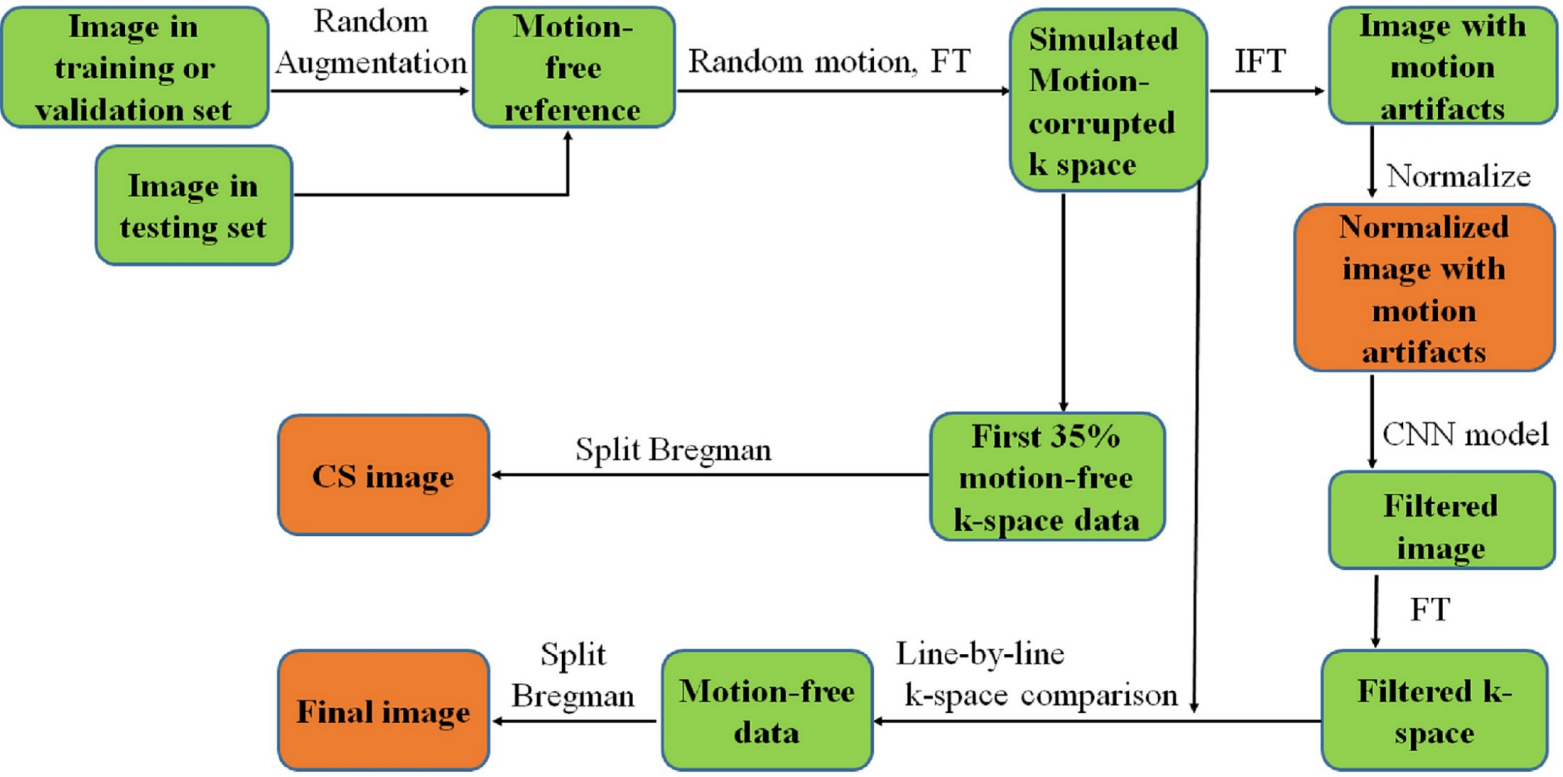
The flowchart of the proposed method.

**Fig 2:**
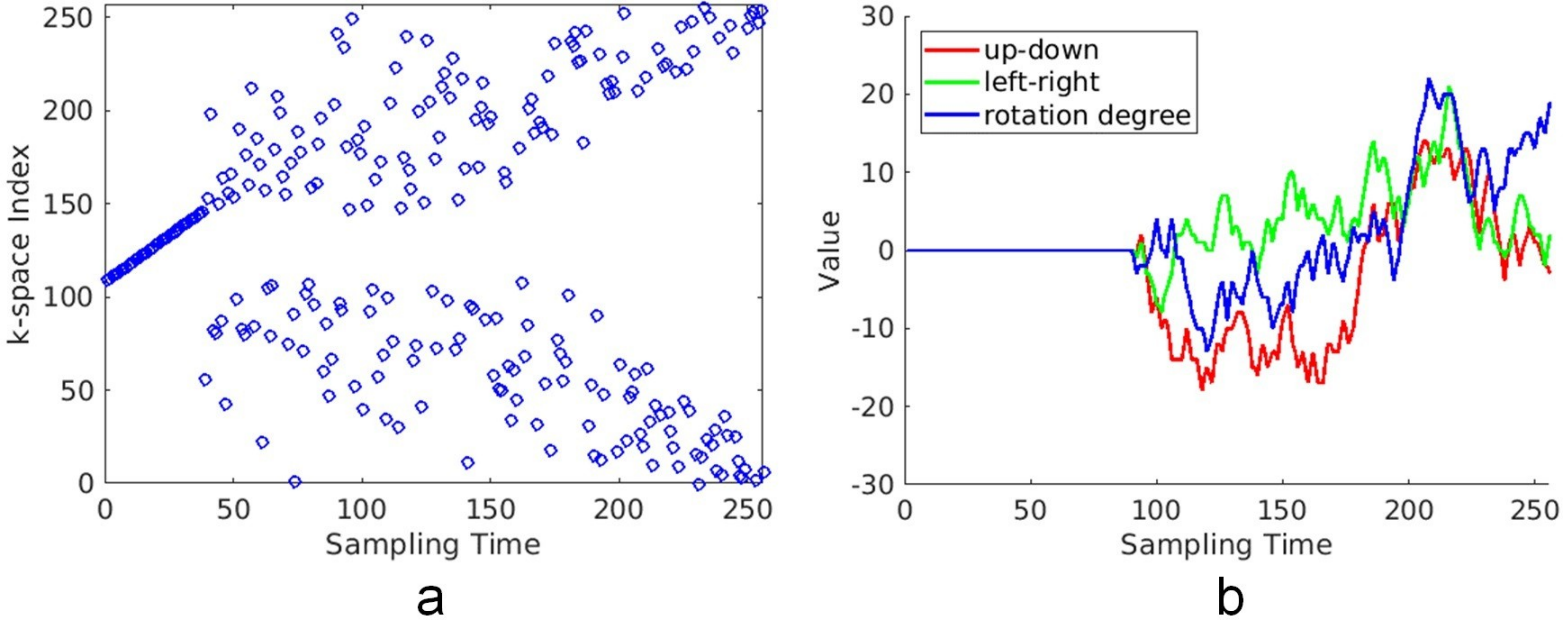
Examples of sampling order and motion track for motion-corrupted image synthesis. (a) An example of the pseudo-random sampling order used in image synthesis. The center 15% of the k-space was sequentially sampled first. Then, the remaining phase-encoding (PE) lines were sampled randomly with a Gaussian distribution, until all 256 lines were sampled. (b) One motion track was used in the simulation. The horizontal axis represents the sampling time, expressed as the number of sampled lines. The vertical axis indicates the amount of translation (unit: pixel) and the rotation (unit: degree). In this motion mode, the object remained still for the first 35% of the total sampling time. Then, a random translation in the range of ±5 pixels and a random rotation in the range of ±5 degrees were applied after each PE line was sampled.

A flowchart of the proposed method is shown in Fig 1. The motion-corrupted k-space (*k*_motion_) was simulated using a pseudo-random sampling order as shown in Fig 2A, and random motion tracks like the one shown in Fig 2B. In the pseudo-random sampling order, 15% of the center part of the k-space was sampled sequentially first, and then the remaining phase-encoding (PE) lines were sampled using a Gaussian distribution. Fig 2B shows a typical example of motion track, in which the motion started after 35% of the k-space had been sampled. The phase encoding lines with motion can be removed in the 2D k-space plane for both 2D and 3D imaging [18], so the motion track was described using three components: the up-down translation (red), the left-right translation (green), and the rotation (blue). Once the motion started, a random translation in the range of -5 to +5 pixels and a random rotation in the range of -5 to +5 degrees were applied after each PE line had been sampled.

Four typical motion modes, in which the motion started at 35%, 40%, 45%, and 50% of the full sampling time, respectively, were simulated and were denoted as M35, M40, M45, and M50, respectively. The simulated k-space data (*k*_motion_) was then inverse-Fourier transformed and normalized to obtain a simulated image with motion artifacts (*I*_motion_) used in this study (as shown in the flowchart in Fig 1).

### Convolutional neural network (CNN) model for filtering images with motion artifacts

**Fig 3:**
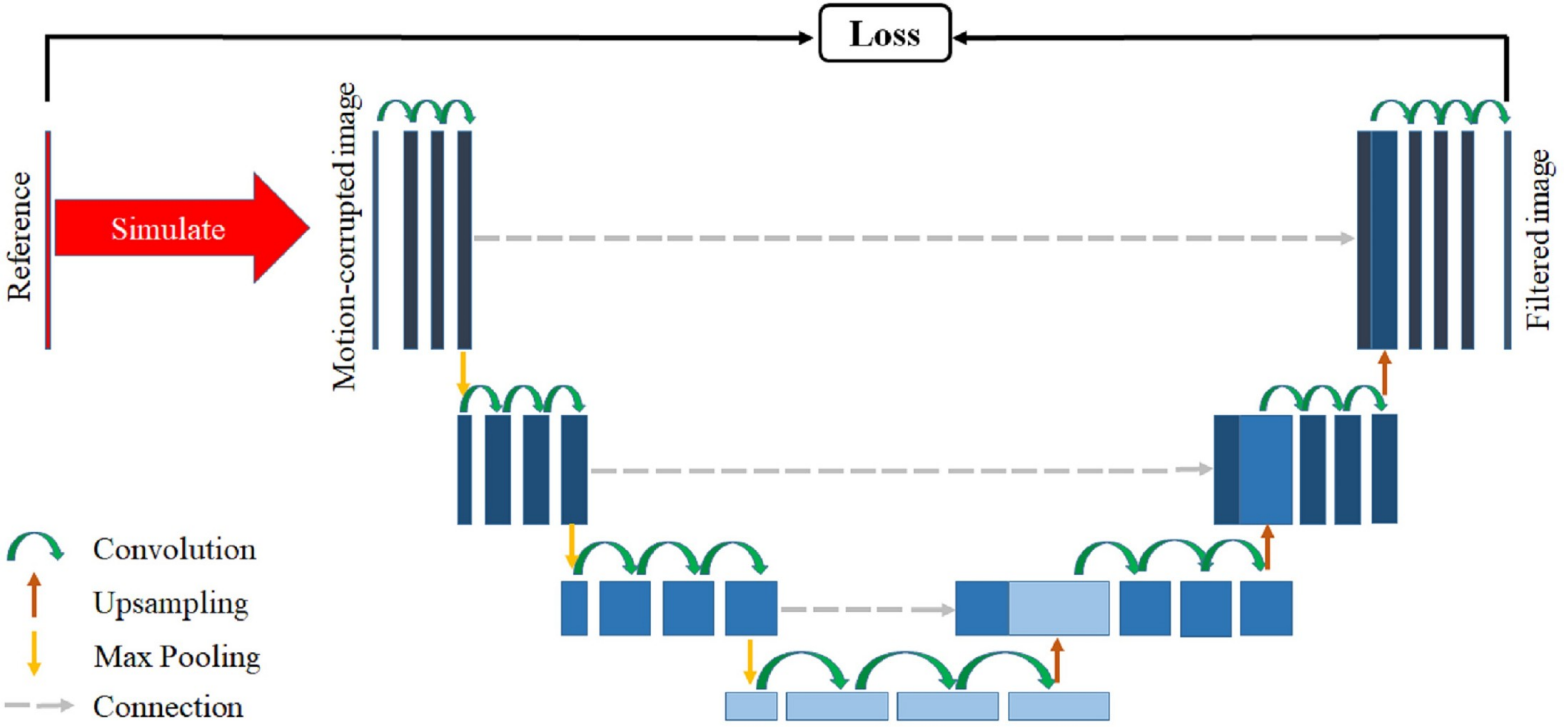
The architecture of the convolutional neural network (CNN) used to filter motion artifacts. The motion-corrupted images and the corresponding reference images were normalized before being used as the inputs of the network. Motion artifacts were alleviated in the output of the network, I_*filtered*_.

First, we trained a CNN model based on U-net [19] to filter the motion-corrupted images to partially remove motion artifacts. The design of our CNN is shown in Fig 3. The model consisted of an encoding path and a decoding path. The encoding path consisted of four repeated blocks, each consisting of two 3 × 3 convolution layers and one 1 × 1 convolution layer. The number of filters for the convolutions were 32, 64, 128, and 256 from top to bottom. Each of these three convolutions was followed by a batch normalization and a Leaky ReLU activation function. Each block was followed by a 2 × 2 max pooling with stride 2 for the down sampling, except for the fourth block. The decoding path was more or less symmetric to the encoding path. Each block in the decoding path consisted of a 2 × 2 transposed convolution (“up-convolution”) for up-sampling, followed by a Leaky ReLU activation, a concatenation with feature maps from the corresponding block in the encoding path, two 3 × 3 convolutions and one 1 × 1 convolution. Each was followed by a batch normalization and a Leaky ReLU activation function. In the last layer, a 1 × 1 convolution was used to produce an output image of the same size as the input image. In total, the network had 25 convolutional layers.

The loss function used in the CNN was as follows:

$$loss=\sum_{i=1}^{\mathrm{batchSize}}\sum_{m=1}^{\mathrm{height}}{\sum_{n=1}^{width}\left\|I_{\mathrm{filtered}}^{\left(i\right)}(m,n)-I_{\mathrm{ref}}^{\left(i\right)}(m,n)\right\|}^{2}$$ (1)

In the above, *batchSize* is the number of images in a batch; *height* and *width* are the height and width of the image, respectively; $I_{filtered}^{\left(i\right)}$ is the i-th output image; and $I_{ref}^{\left(i\right)}$ is the corresponding motion-free reference image. During training, the Adam optimizer was used. Maximum number of epochs number was set to 200. All the training images were shuffled before each epoch, and each epoch had 650 steps with batchSize of 10. The initial learning rate was set to 0.001, and it was multiplied by 0.5 whenever the validation loss did not decrease for 10 consecutive epochs. Early stopping was used if the validation loss did not decrease for 50 epochs.

When the CNN model was trained, the normalized images of *I*_motion_ were simulated with motion mode M35, and the corresponding *I*_ref_ images were used as inputs. Random translation (±10 mm), rotation (±5 degrees), scaling (0.1), shearing (0.1) and horizontal flip were applied to the original images in the training and validation sets, to get the augmented motion-free images *I*_ref_. The *I*_ref_ images were then used to simulate the *I*_motion_ images using the above-mentioned approach. After the CNN model was trained, the simulated images *I*_motion_ in the test set were filtered to get the filtered images *I*_filtered_. The above training, validation, and test were all implemented in Python 3.6.5, using the GPU version of TensorFlow 1.13.1 and Keras 2.3.1, based on Ubuntu 18.04 and NVIDIA TITAN Xp.

### Detection of lines influenced by motion in k-space

After the CNN model filtered *I*_motion_ and produced *I*_filtered_, a Fourier transform (FT) was applied to *I*_filtered_ to get the corresponding k-space *k*_filtered_. Then, we compared *k*_filtered_ with *k*_motion_ line-by-line for all PE lines, and calculated PSNR_k_ that was rewritten according to the form of peak signal-to-noise ratio (PSNR) as follows:

$$\mathrm{PSN}R_{k}(n)={10\log}_{10}{\left({MAX}_{k}(n\right)}^{2}/{MSE}_{k}(n))$$ (2)

And

$${MAX}_{k}(n)=\max(|k_{\mathrm{motion}}(m=1\ldots \mathrm{numR},n)|)$$ (3)

$${MSE}_{k}(n)=(\sum_{m=1}^{numR}{\left(|k_{\mathrm{motion}}(m,n)‐k_{\mathrm{filtered}}(m,n)|\right)}^{2})/\mathrm{numR}$$ (4)

In the above Formulas (2), (3) and (4), n is the index of the PE line, PSNR_k_(*n*) is the PSNR_k_ for the nth PE line. The function max(set) was to get the maximum of the set. numR is the number of read-out encoding in each line. According to the Formulas (2), (3) and (4), the closer the values of two PE lines were, the higher their PSNR_k_. We used Otsu’s method [20] to classify the calculated PSNR_k_ into two groups. The group with the higher PSNR_k_ values consisted of the PE lines not affected by motion. To find the exact time when the motion started, we arranged all of the k-space lines in the order of sampling, and used 0/1 to represent every line whose PSNR_k_ was above/below the threshold, respectively, to get a sequence similar to the following:

0000000000…001000011111011111…

Here, each 0 represents a k-space line not affected significantly by the motion and each 1 represents a k-space line affected by the motion. To make the detection more robust to noise, we first excluded from the sequence those lines in the high-frequency region of the k-space (40 lines on either side of k-space), where the Signal to noise ratio (SNR) was low. We treated two consecutive 1s in the sequence as the start of the motion. All data sampled before this point were then used for the final image reconstruction.

### Reconstruction of final images and statistical analysis

After the motion-free PE lines were detected, we employed the split Bregman algorithm to perform CS reconstruction, using these lines as under-sampled data to get the final images. The mean and standard deviation (SD) of PSNR and structural similarity (SSIM) were used to quantitatively evaluate the final images. The mean and SD of PSNR and SSIM values for the filtered images and the CS reconstructions using fixed 35% under-sampled data were also calculated and compared with those of the proposed algorithm. One-sample Kolmogorov-Smirnov (KS) tests were used to determine if the distributions of the values of both PSNR and SSIM in each group of images conformed to normal distributions or not. If meeting normal distributions, the performances of different models and methods were compared using t-tests, otherwise using Two-sample KS tests, and a P value < 0.05 was considered statistically significant. The statistical analysis was implemented by Python 3.6.5 and SPSS Statistics 22.

## Results

### Motion detection by k-space analysis

**Fig 4:**
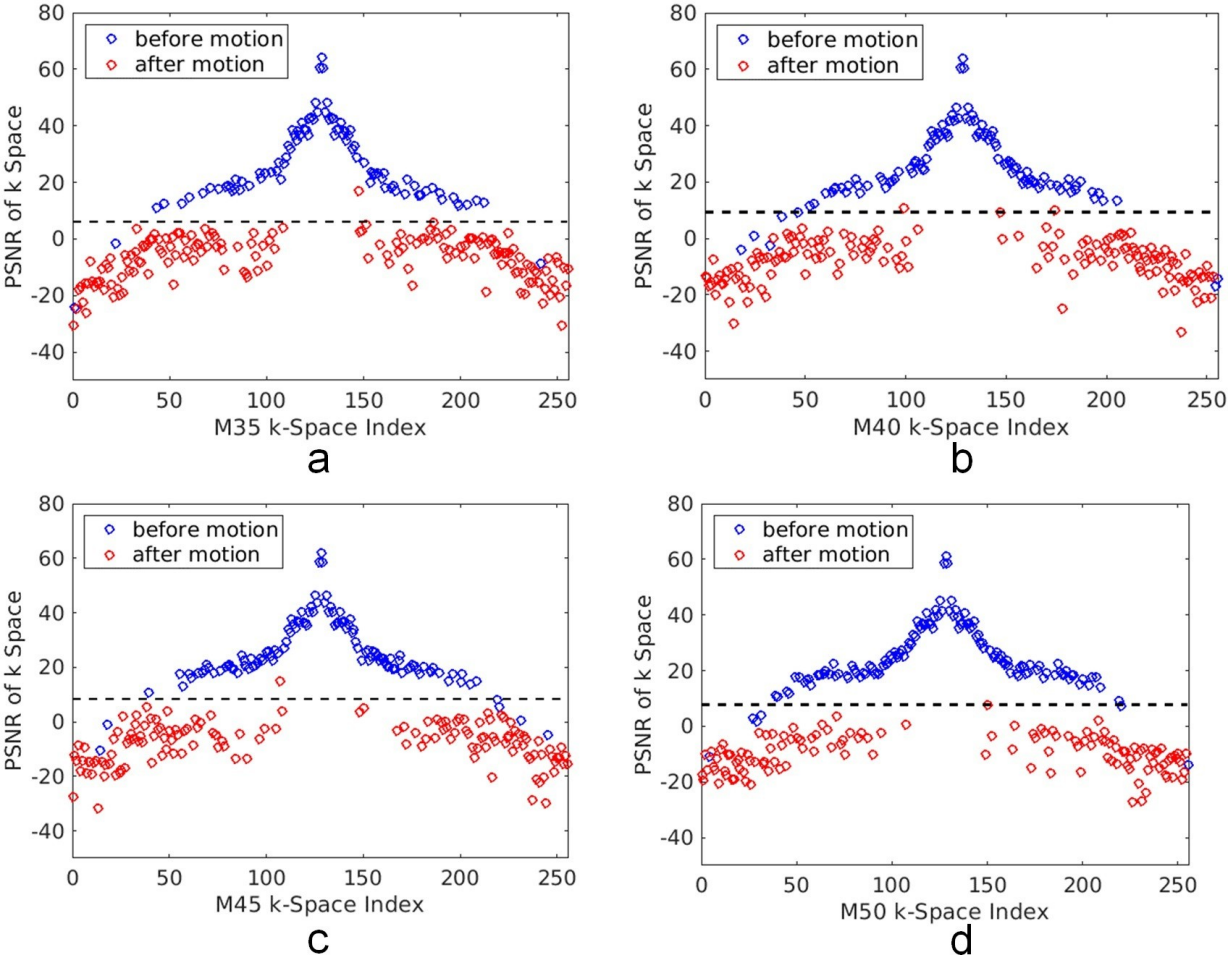
The examples of PSNR_k_ between the PE lines of the motion-corrupted k-space and filtered k-space for each of the four motion modes. (a)–(d) show the calculated PSNR_k_ values for M35, M40, M45 and M50, respectively. Blue circles and red circles represent the PE lines sampled before and after the motion started, respectively. The horizontal dashed line visualizes the threshold determined by Otsu’s method for splitting the calculated PSNR_k_ values into two groups. It can be seen most of the lines not affected by the motion stay above the threshold, except for a few lines in the high-frequency region.

For each test image, we calculated the PSNR_k_(*n*) between the PE lines of the motion-corrupted k-space and filtered k-space for all motion modes, namely, M35, M40, M45 and M50. Then, Otsu’s method was used to classify the PSNR_k_ of each image into two groups. The results for one typical image are shown in Fig 4. From Fig 4, it can be seen clearly that most PE lines not affected by motion can be distinguished from those affected by motion, except for those in the high-frequency region of the k-space.

**Fig 5:**
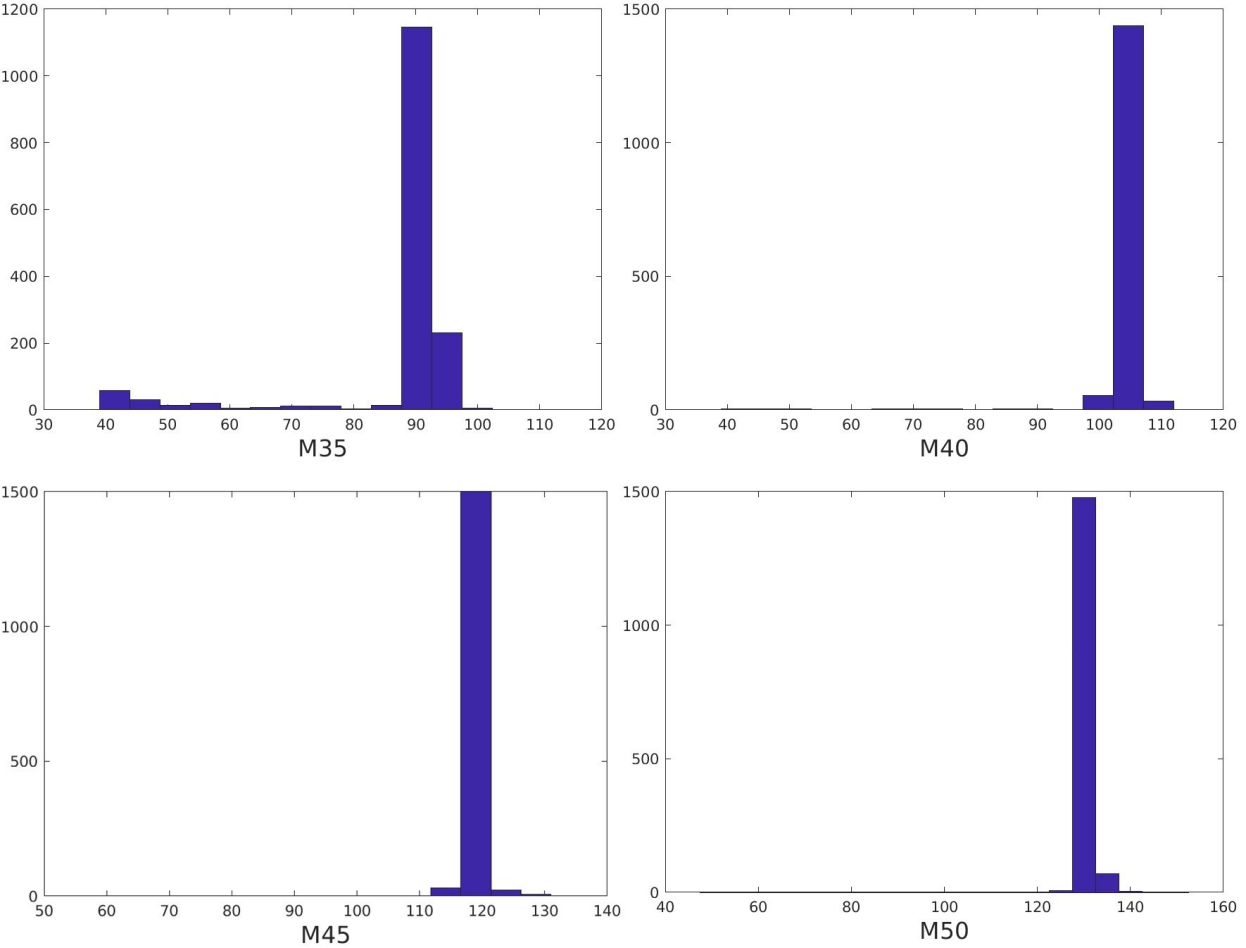
Histograms of the detected motion time for the four motion modes for all of the images in the test set.

For each motion mode, we used the above algorithm to detect the motion start time for all of the images in the test set. Fig 5 shows the histograms of the detected motion time for the four motion modes. From the histograms, we can see clearly that for most of the images, the motion start time could be detected quite accurately (as shown by the peak at approximately 103 for M40). As seen in Table 1, for 65.90%, 73.72%, 75.58% and 73.97% of all the test image slices, the motion detection was 100% accurate.

**Table 1:** Results of motion detection.

Motion Mode	Percentage of perfect motion detection	Average of motion detection error in k-space lines
M35	65.90	13.60
M40	73.72	4.68
M45	75.58	2.13
M50	73.97	2.06

The percentages of perfect motion detection for four motion modes (M35, M40, M45 and M50), and their corresponding averages of motion detection error in k-space lines respectively.

### Final images and comparison with filtered images and direct CS reconstruction

**Fig 6:**
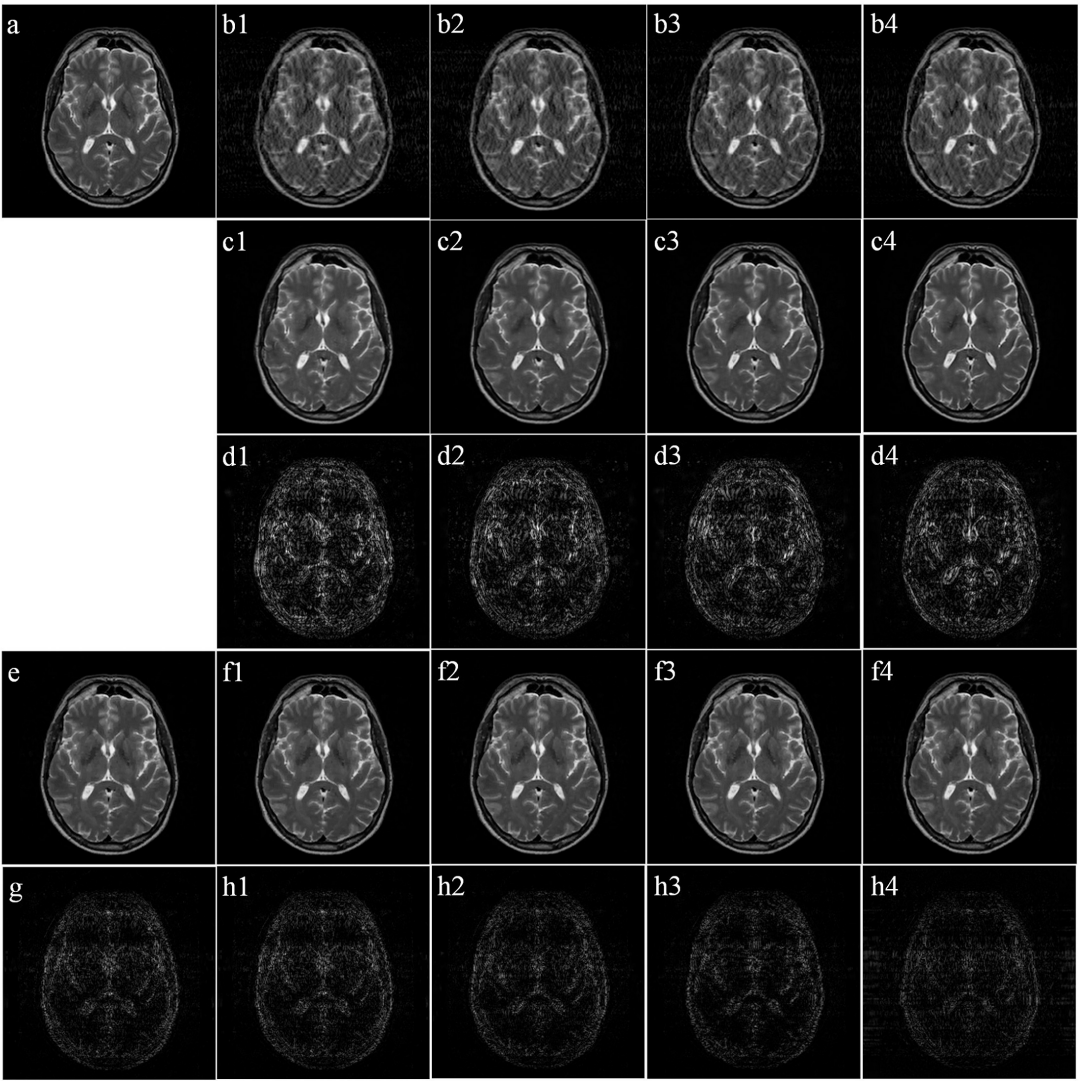
Final images reconstructed using the proposed algorithm. (a) Reference image without motion; (b1), (b2), (b3), and (b4) are the simulated motion-corrupted images for motion mode M35, M40, M45, and M50, respectively; (c1), (c2), (c3), and (c4) are the filtered images by trained CNN model for (b1), (b2), (b3) and (b4), respectively; and (d1–d4) are the corresponding difference images (5×) between images (c1–c4) and the gold standard (a). (e) Image reconstructed using CS directly using 35% under-sampled data; (f1), (f2), (f3), and (f4) are the images constructed using the proposed method for M35, M40, M45, and M50, respectively; and (g, h1–h4) are the corresponding difference images (5×) between images (e, f1 –f4) and the gold standard (a).

After detecting when the motion started, all lines sampled before this were regarded as not affected by motion and used in the final CS reconstruction. The examples of final reconstructed images for the four motion modes are shown in Fig 6. From Fig 6, it can be seen that the quality of the final images are obviously better than those filtered images, with no apparent motion artifacts present. For motion mode of M35, the quality of the image reconstructed by the proposed method is comparable to the image reconstructed by CS directly using unaffected under-sampled data with a fixed sampling ratio of 35%. However, in the three other motion modes, with the delay of the motion, an increasing number of unaffected PE lines were detected and used in the CS reconstruction, making the quality of final reconstructed images increasingly better. These results were also supported by the statistical results, as shown in Tables 2 and 3. The distributions of the values of both PSNR and SSIM in each group of images did not conform to normal distributions after One-sample KS tests (*P*<0.001, the results of One-sample KS test in Table 2), so performances of different models and methods were compared using Two-sample KS tests. In each motion mode, both the mean PSNR and mean SSIM values of the final images were all significantly higher than the filtered images (P<0.001, the results of One-sample KS test in Table 2 and the results of Two-sample KS test in Table 3). Compared with CS using 35% data, the proposed algorithm produced images of higher PSNR and SSIM values (*P* < 0.001, the results of One-sample KS test in Table 2 and the results of Two-sample KS test in Table 3) except for motion mode M35.

**Table 2:** The mean and standard deviation (SD) of peak signal-to-noise ratio (PSNR) and structural similarity (SSIM) after one-sample Kolmogorov-Smirnov (KS) test.

	Still lines	PSNR (mean±SD; Asymp.Sig.)	SSIM (mean±SD; Asymp.Sig.)
Corrupted	35%	29.414±3.003; P<0.001	0.718±0.068; P<0.001
	40%	30.375±3.057; P<0.001	0.742±0.064; P<0.001
	45%	31.596±3.025; P<0.001	0.771±0.059; P<0.001
	50%	33.018±3.051; P<0.001	0.802±0.054; P<0.001
Filtered	35%	32.820±2.788; P<0.001	0.916±0.044; P<0.001
	40%	33.840±2.767; P<0.001	0.924±0.041; P<0.001
	45%	34.531±2.648; P<0.001	0.927±0.039; P<0.001
	50%	34.762±2.595; P<0.001	0.925±0.038; P<0.001
CS	35%	37.678±3.261; P<0.001	0.964±0.028; P<0.001
Final	35%	36.129±3.678; P<0.001	0.950±0.046; P<0.001
	40%	38.646±3.526; P<0.001	0.964±0.035; P<0.001
	45%	40.426±3.223; P<0.001	0.975±0.025; P<0.001
	50%	41.510±3.167; P<0.001	0.979±0.023; P<0.001

After the one-sample KS test, the mean and SD of PSNR and SSIM for the motion-corrupted images, filtered images, images by compressed sensing (CS) with a sampling ratio of 35%, and final images constructed by the proposed algorithm were presented respectively, and the two tailed asymptotic significances (Asymp.Sig.) all had P<0.001 witch indicated not conforming to normal distribution.

**Table 3:** Performance of comparison with two-sample KS test.

Comparison		PSNR (P, two tailed)	SSIM (P, two tailed)
Between filtered images and images by CS with a sampling ratio of 35%	35%	P < 0.001	P < 0.001
	40%	P < 0.001	P < 0.001
	45%	P < 0.001	P < 0.001
	50%	P < 0.001	P < 0.001
Between final images and images by CS with a sampling ratio of 35%	35%	P < 0.001	P < 0.001
	40%	P < 0.001	P < 0.001
	45%	P < 0.001	P < 0.001
	50%	P < 0.001	P < 0.001
Between final images and filtered images in four motion modes	35%	P < 0.001	P < 0.001
	40%	P < 0.001	P < 0.001
	45%	P < 0.001	P < 0.001
	50%	P < 0.001	P < 0.001

Performance of comparison between filtered images and images by compressed sensing (CS) with a sampling ratio of 35%, between images by the proposed algorithm and images by CS with a sampling ratio of 35%, between final images constructed by the proposed algorithm and filtered images in four motion modes, respectively. Two-sample KS test was used for each comparison, its P value of two tailed test was presented in the table, and a P < 0.05 was considered to indicate a statistically significant difference in performance. So each comparison had significant difference (P<0.001) as showed in the table.

## Discussion

The CNN model trained in this study mapped the motion-corrupted images to the corresponding motion-free images. It was essentially an image filter for motion artifact mitigation. Although the quality of the filtered image was not optimal, it was closer to the gold standard motion-free image than the motion-corrupted image. Therefore, the filtered k-space data was more like the k-space of the reference image.

From the process of image simulation, we knew that for PE lines not affected by motion, the data in *k*_motion_ was exactly the same as the data in *k*_ref_. Therefore, it could be expected that *k*_motion_ and *k*_filtered_ were more similar for those PE lines. For the same reason, the difference between *k*_motion_ and *k*_filtered_ should have been greater on the PE lines affected by motion. Therefore, we could find out which PE lines were affected by motion by comparing *k*_motion_ and *k*_filtered_ line-by-line.

This was the basic approach in this study, i.e., using filtered k-space data to detect the k-space PE lines affected by motion. It was also the main difference between our method and a recent study [14] that used a combined CNN and model-based method to detect corrupted k-space encoding lines. In Fig 4, it was shown clearly that our proposed method can accurately identify most PE lines affected by motion, except for the data with a poor SNR in the high-frequency region of the k-space. Moreover, the quality of the final images reconstructed by the proposed method was improved as indicated by the quantitative metrics such as PSNR and SSIM.

Deep learning has also been used to detect motion artifacts in MRI images. Kustner T, et al. [11] proposed to use CNN to derive probability maps for the presence of motion artifacts from image patches. Sreekumari A, et al. [12] developed a deep learning approach to determine whether a series need to be rescanned. Meding K, et al. [13] used CNN to implement fully automated detection of motion artifacts in MRI. All these studies used deep learning to detect the existence of motion artifacts in MR images and can be used as quality assurance approaches. However, they could not determine which part of k-space data was affected by motion, so all the collected data had to be abandoned whenever a rescan was required. In contrast, we used k-space analysis following a CNN-based image filtering to find the k-space data not affected by motion and used them to reconstruct the final image, thus avoided a rescan in most cases.

Deep learning has also been used to remove motion-artifacts in MRI images. Pawar K, et al. [21] used Inception-ResNet to suppress motion artifacts in MRI. Johnson PM, et al. [15] used DCNN as a generator of cGAN to remove motion artifacts and found that while substantial artifact suppression was achieved, the generated images appeared slightly oversmoothed. Tamada D, et, al [22] proposed a CNN-based method (MARC) to remove motion artifacts caused by respiratory motion in images obtained via dynamic contrast enhanced MRI. Duffy BA, et al. [16] proposed to use CNN to correct motion-corrupted images and suggested use adversarial loss together with L1 loss to improve the algorithm. Kustner T, et al. [23] also demonstrated that deep learning-based algorithm can retrospectively remove motion artifacts and generate near-realistic motion-free images. Armanious K, et al. proposed a MedGAN with a U-net based generator and produced near-realistic MR images from severely deteriorate scans [17]. While all these approaches could suppress motion artifacts and improved the image quality, they all suffered from image over smoothing and failure to keep anatomical details and diagnostic information. Therefore, their clinical applicability needs to be confirmed. In contrast, we used only data unaffected by motion to reconstruct the final image with CS. Since no motion corrupted data was present in the raw data, the reconstruction will be much easier. Furthermore, CS has already been widely adapted in commercial MRI scanners, which demonstrated its ability to reconstruct high quality MR images that meet the requirement of clinical use from under-sampled k-space data.

There are also many more traditional approaches to deal with motion artifacts in MR images. Among them, the most straightforward ones are fast imaging. For example, single-shot imaging [1] and echo planar imaging (EPI) [24] can both shorten the acquisition time, and thus reduce the probability of motion. However, they also have their limitations: single-shot imaging has limited spatial resolution [1], and EPI is vulnerable to geometric distortions and chemical-shift artifacts [1]. Parallel imaging methods [25], such as "SENSE", and "GRAPPA", can be combined with various pulse sequences to accelerate image acquisition. They require special configurations of coils, and if motion occurs during the acquisition of the reference data, new types of artifacts will be generated [25–27].

Typical retrospective methods use special pulse sequences to collect positional-related information to be used in image construction. For example, MR navigator [28, 29] is often used to obtain data related to the position of the object scanned and to detect movements. However, the additional coding required increases the acquisition and processing time. The "periodically-rotated overlapping parallel lines with enhanced reconstruction" approach [30, 31] samples the center of the k-space repeatedly and uses the redundant data to make it robust to motion, but it uses non-Cartesian sampling, which may introduce reconstruction-related artifacts.

Prospective correction methods [32–35] adaptively adjust the scanning coordinates to keep the spatial position and orientation of the object unchanged in the scanning coordinates. These require external markers and tracking systems to track the motion of a patient and to adjust sequence parameters accordingly in real time, which makes it the hardest to implement.

Our proposed method is also retrospective. It is similar to the navigator method, in the sense that they both use k-space data to detect motion. However, the proposed method directly uses the k-space data for motion detection, thus it saves acquisition time and requires no major modification of the pulse sequence. Integrating this approach into a pulse sequence only requires some minor modifications of the sequence, e.g., to use a pseudo-random sampling order for data acquisition. Thus, it can be readily integrated into various pulse sequences, including some fast imaging sequences.

Most traditional post-processing correction techniques either assume a specific movement during the scanning process (e.g., using navigator data) is known, or involve an iterative algorithm to optimize image entropy, gradient entropy, and other artifact measures to achieve the correction [36–38]. In contrast, we used a deep learning model as an image filter, and directly compared k-space PE lines to sift the data affected by motion. This approach was simple in concept, and involved the hand-tuning of only a few parameters. Thus, the proposed approach can be easily extended to work with different pulse sequences of different body parts. It was also time-efficient in calculation, as it did not use iterative optimization except for in the split-Bregman algorithm, which proved to be fast enough for typical image reconstruction [3]. To further accelerate the reconstruction process, another CNN model can be used to replace the split-Bregman algorithm [10].

The proposed method also has some limitations. First, minor modifications should be applied to pulse sequences to use the pseudo-random sampling order required by our method. In addition, it requires that the subjects remain still in the early stage of sampling, to ensure high quality CS reconstruction, though it is easier for most subjects to keep still in the start of the scan. The advantage of the proposed approach over CS acquisition with a fixed sampling ratio is that when the subjects can remain still for longer, more data can be used to reconstruct images of higher quality. Secondly, the CNN model used to filter the motion-corrupted images was trained with only simulated images from motion mode M35, but it can still filter the simulated images with other motion modes. It demonstrated CNN’s good ability of generalization. However, for images with different contrast acquired with different pulse sequences, it is preferable to train specific models for specific pulse sequences.

## Conclusion

By comparing the k-space of an image filtered by a CNN model and a motion-corrupted k-space, we could accurately determine the PE lines affected by motion and exclude them from the final CS reconstruction, thereby producing high-quality MR images without motion artifacts. Experiments on simulated data prove that this method can effectively alleviate motion artifacts. This approach imposes no hardware requirements and requires only minor modifications of the pulse sequences. More schemes can also be designed to make use of the general idea in this approach. For example, to improve the accuracy of motion detection, PE lines in the low or intermediate frequency region of the k-space can be scanned repeatedly during the scanning, as probes for motion detection. Redundant data in these k-space can also be used to improve the image SNR in image reconstruction. Thus, we expect this approach to provide a new alternative for elimination of motion artifacts in MRI.
